# The Magnitude of Diving Bradycardia During Apnea at Low-Altitude Reveals Tolerance to High Altitude Hypoxia

**DOI:** 10.3389/fphys.2019.01075

**Published:** 2019-08-22

**Authors:** Pontus Holmström, Eric Mulder, Angelica Lodin Sundström, Prakash Limbu, Erika Schagatay

**Affiliations:** ^1^Department of Health Sciences, Mid Sweden University, Sundsvall, Sweden; ^2^Department of Nursing Sciences, Mid Sweden University, Sundsvall, Sweden; ^3^Department of Clinical Physiology, Nepalese Army Institute of Health Sciences, Kathmandu, Nepal

**Keywords:** acute mountain sickness, breath-hold diving, hypoxia, prediction, cardiovascular diving response, spleen

## Abstract

Acute mountain sickness (AMS) is a potentially life-threatening illness that may develop during exposure to hypoxia at high altitude (HA). Susceptibility to AMS is highly individual, and the ability to predict it is limited. Apneic diving also induces hypoxia, and we aimed to investigate whether protective physiological responses, i.e., the cardiovascular diving response and spleen contraction, induced during apnea at low-altitude could predict individual susceptibility to AMS. Eighteen participants (eight females) performed three static apneas in air, the first at a fixed limit of 60 s (A1) and two of maximal duration (A2–A3), spaced by 2 min, while SaO_2_, heart rate (HR) and spleen volume were measured continuously. Tests were conducted in Kathmandu (1470 m) before a 14 day trek to mount Everest Base Camp (5360 m). During the trek, participants reported AMS symptoms daily using the Lake Louise Questionnaire (LLQ). The apnea-induced HR-reduction (diving bradycardia) was negatively correlated with the accumulated LLQ score in A1 (*r*_*s*_ = −0.628, *p* = 0.005) and A3 (*r*_*s*_ = −0.488, *p* = 0.040) and positively correlated with SaO_2_ at 4410 m (A1: *r* = 0.655, *p* = 0.003; A2: *r* = 0.471, *p* = 0.049; A3: *r* = 0.635, *p* = 0.005). Baseline spleen volume correlated negatively with LLQ score (*r*_*s*_ = −0.479, *p* = 0.044), but no correlation was found between apnea-induced spleen volume reduction with LLQ score (*r*_*s*_ = 0.350, *p* = 0.155). The association between the diving bradycardia and spleen size with AMS symptoms suggests links between physiological responses to HA and apnea. Measuring individual responses to apnea at sea-level could provide means to predict AMS susceptibility prior to ascent.

## Introduction

Recreational trekking has become a popular sporting activity, and approximately 40 million people travel to high altitude (HA; ≥3000 m) each year (Neupane and Swenson, 2017). As both the individual rate of acclimatization (Schoene et al., 1984; Gallagher and Hackett, 2004) as well as the individual susceptibility to acute mountain sickness (AMS) varies greatly (Oliver et al., 2012), the prediction of individual susceptibility is imperative to reduce AMS incidence.

Several studies have previously investigated whether individual susceptibility to AMS can be predicted. These studies include a wide range of physiological markers, but with inconsistent findings. For example, Roach et al. (1998) found an association between AMS and SaO_2_, at 4200 m, wherein participants with SaO_2_ ≥ 84% were at less risk of developing AMS. In addition, Karinen et al. (2010) compared resting SaO_2_ and post-exercise SaO_2_ during ascent to 5300 m with the subsequent development of AMS. At 3500 and 4300 m both resting SaO_2_ and post-exercise SaO_2_ were lower in participants who developed AMS at higher altitudes. Conversely, some researchers have failed to find any association between AMS and SaO_2_ at altitude (Wagner et al., 2012).

Some investigators have found an association between both the hypoxic ventilatory response (HVR) and the hypercapnic ventilatory response (HCVR) with AMS (Nespoulet et al., 2012; Richalet et al., 2012). Nespoulet et al. (2012) measured carbon dioxide (CO_2_) and oxygen (O_2_) chemo-sensitivity during 20 min hypoxic eupnea in AMS susceptible individuals, during which both variables were found to be reduced in symptomatic compared to asymptomatic individuals. Others have not found any association between AMS and HVR (Milledge et al., 1991; Hohenhaus et al., 1995). For example, Hohenhaus et al. (1995) reported no difference in HVR and HCVR between AMS susceptible and symptom-free individuals, during isocapnic hypoxia.

Furthermore, short apneic duration at sea-level has been found to be an independent predictor of AMS (Austin and Sleigh, 1995). There was also an association between the gag reflex and AMS symptoms (Austin and Sleigh, 1995), and Hayat et al. (2006) found an association between hyperventilation capacity and development of AMS, indicating that general respiratory control may lead to lower risk of developing AMS. Moreover, Karinen et al. (2012) found that heart rate (HR) variability at altitude was associated with development of AMS, however, others have reported no association (Boos et al., 2017). Therefore, despite all past work, to date AMS susceptibility cannot be accurately predicted at sea-level prior to ascent.

Apneic diving naturally induces hypoxia, which initiates several protective responses. The cardiovascular diving response (CVD) is initiated by apnea which is enhanced by facial cooling and serves as the first line of defense against hypoxia during apnea, which occurs via sympathetic outflow to peripheral blood vessels leading to vasoconstriction and by parasympathetic outflow to the heart inducing bradycardia (Gooden, 1994; Andersson and Schagatay, 1998). The diving bradycardia slows the depletion of O_2_ during apnea, which extends the apnea duration. This response is more pronounced in professional apneic divers compared to non-divers (Schagatay and Andersson, 1998). The CVD is often quantified by HR-reduction, with reductions typically between 15 and 40% (Alboni et al., 2011). As a second line of defense against hypoxia during apnea, spleen contraction occurs which leads to a transient increase in circulating hematocrit (Hct) and hemoglobin concentration (Hb) and prolonged apneic duration (Schagatay et al., 2001; Bakovic et al., 2003). Expert apneic divers have been found to have exceptionally large spleens (Schagatay et al., 2012).

Accordingly, apneic diving and exposure to altitude are two naturally occurring activities in humans, possibly sharing protective mechanisms against an hypoxic insult. A recent study found that individuals with a pronounced diving response during maximal apnea also maintained higher SaO_2_ at simulated altitude (Starfelt et al., 2018). To evaluate the effects of low-altitude, relative to sea-level, on individual apnea-induced physiological responses, a pilot study was conducted in normobaric hypoxia, which showed that the physiological responses remain consistent between sea-level and simulated low-altitude conditions, without prior acclimatization (Holmström et al., 2018). Based on these promising results, our aim was to investigate if an apneic test that naturally induces hypoxia at sea-level could predict individual susceptibility to AMS during an expedition to HA. We hypothesized that the apnea-induced HR-reduction (diving bradycardia) and spleen contraction would be negatively associated with AMS symptom score in a Lake Louise Questionnaire (LLQ) used during a HA trek.

## Materials and Methods

### Participants

Twenty-two participants, who were already participating in a trekking expedition to mount Everest base camp (EBC; 5360 m), volunteered to participate in the Study. Included in the final analysis were 18 participants; 8 females and 10 males (mean ± SD age was 44 ± 14 years; height: 174 ± 8 cm; weight: 72 ± 12 kg; and vital capacity [VC]: 4.8 ± 0.8 L). Four participants were excluded for taking Acetazolamide which is used to prevent and reduce symptoms of AMS.

Twelve participants had visited HA (≥3000 m) previously, but none in the previous 2 months. Thus, the participants were recreational un-acclimatized trekkers with no prior experience in apneic diving. Human ethical review boards in Sweden and Nepal approved test protocols and all participants gave written informed consent to participate in accordance with the Declaration of Helsinki.

### Procedures

The experimental procedure consisted of two parts: (1) an apnea test conducted in Kathmandu, Nepal, at an altitude of 1400 m and (2) a high altitude trek to EBC.

The participants were asked to report to the lab after at least 1 h without eating or drinking caloric beverages. They entered the lab directly after climbing stairs up to the fourth floor, their height and weight was measured, and they were seated to rest on the chair where the apnea test was conducted and they signed a consent form. Before the apnea test started, after 2 min of seated rest, baseline VC (L) was recorded in duplicate (Vitalograph, Ltd., Compact II, Buckingham England) and the larger volume used for analysis. During the following rest, they received written and oral instructions about the test and after a minimum of 10 min rest, a 2-min countdown for the apnea test started.

#### Apnea Test

**FIGURE 1:**
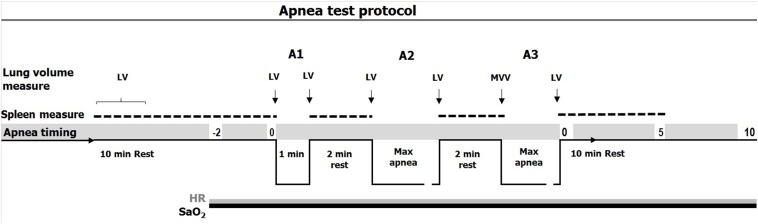
Timing of apneas (A1: apnea 1; A2: apnea 2; A3: apnea 3) with spleen measure (doted line), continuous measure of SaO2 (arterial oxygen saturation, black line) and HR (heart rate, gray line) and lung volume measures. 15 s of hyperventilation preceded apnea 3 (A3) combined with a MVV (maximal voluntary ventilation) test.

The test consisted of three consecutive static apneas in air, with the first at a fixed time limit of 1 min: apnea one (A1), followed by two maximal voluntary duration apneas: apnea two (A2) and apnea three (A3), all of which were separated by 2 min rest (Figure 1; Engan et al., 2014). While normal breathing preceded the first two apneas, participants were instructed to hyperventilate for 15 s before A3, combined with a maximal voluntary ventilation (MVV) test. Participants were handed a nose-clip at 30 s before apneas and notified when 10 s remained and a second by second countdown was initiated.

Participants were instructed to exhale fully and take a deep but not maximal breath preceding each apnea, while inspiratory volume was measured. Participants were told to keep the chest relaxed and refrain from swallowing or exhaling during apneas. If SaO_2_ levels decreased below 60% during apneas, the participants would be told to resume breathing in order not to risk syncope, which may occur below 50% SaO_2_. Upon termination of apnea, the nose-clip was removed, and the participants were instructed to resume normal breathing. Apneic duration was monitored with a stopwatch.

##### Measurements

After 5 min of rest, blood pressure, and forehead temperatures were measured in duplicate to exclude hypertension (200/100) or fever (>38°C). The spirometer were also used for inspiratory and expiratory volumes as well as MVV measurements in the apnea tests. SaO_2_, HR, partial pressure of end-tidal CO_2_ and respiratory rate were continuously logged throughout the apnea test, using a combined pulse oximeter-capnograph (Medair Lifesense LS1-9R, Medair AB, Delsbo, Sweden) attached to the middle finger, from two min prior to A1 until 10 min after A3 (Figure 1). Data were stored via memory unit (Trendsense, Nonin Medical, Inc., Medair AB, Hudiksvall, Sweden). Spleen size was measured via ultrasonic imaging (M-Turbo Ultrasound system, FUJIFILM SonoSite, Inc., Bothell, WA, United States) each minute for 10 min during the rest period and immediately after the termination of each apnea, during which measurements were taken each minute from the end of each apnea until 10 min after A3 (Figure 1). During each minute, three maximal spleen measures were recorded (length, thickness, and width).

#### High Altitude Trek

**FIGURE 2:**
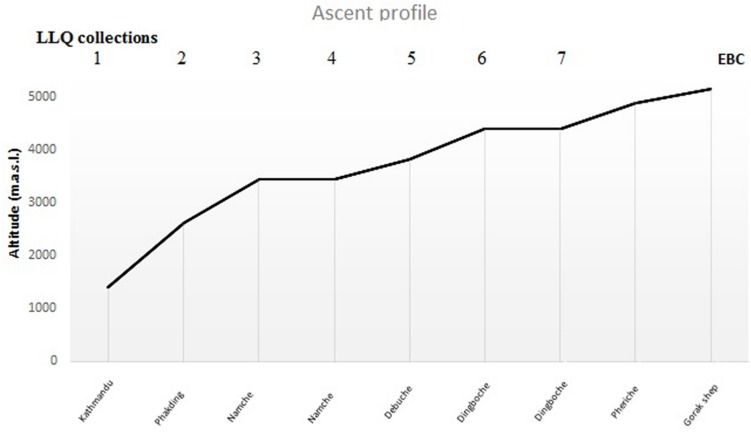
Sleeping altitudes for data collection of Lake Louise Questionnaire (LLQ) score (days 1–7) included in the analysis during ascent to EBC (*n* = 18).

The morning after the apnea test, the participants flew to Lukla (2840 m) and started the EBC trek. They hiked in three groups, organized by a local trekking agency and followed the same ascent profile to Dingboche (4410 m; Figure 2) reaching Gorak Shep (5160 m) on the 9th day. The trek included two rest days, and arrived back in Kathmandu on the morning of the 14^*th*^ day. At least one experienced Sherpa guide led each group. During the ascent, the participants documented their AMS symptoms in a booklet, each morning using the LLQ. The participants had been thoroughly informed on how to use the LLQ booklet in conjunction with the apnea test. SaO_2_ and HR was also measured each morning with a portable pulse oximeter (Nonin Medical, Inc., Medair AB, Delsbo, Sweden), which was supervised by the guides. After the hike, participants returned the LLQ booklet to the research staff.

##### Lake Louise Questionnaire

The LLQ is a self-administered questionnaire consisting of five items quantifying the presence of the most frequent AMS symptoms, including: (1) headache, (2) gastrointestinal symptoms, (3) fatigue, (4) dizziness, and (5) difficulty sleeping. Each item is graded on a scale from 0 (no symptom) to 3 (severe symptom) as described by Roach et al. (1993). In addition, medication taken during the trek and morning values of SaO_2_ and HR were noted in the LLQ booklet.

### Analysis

**FIGURE 3:**
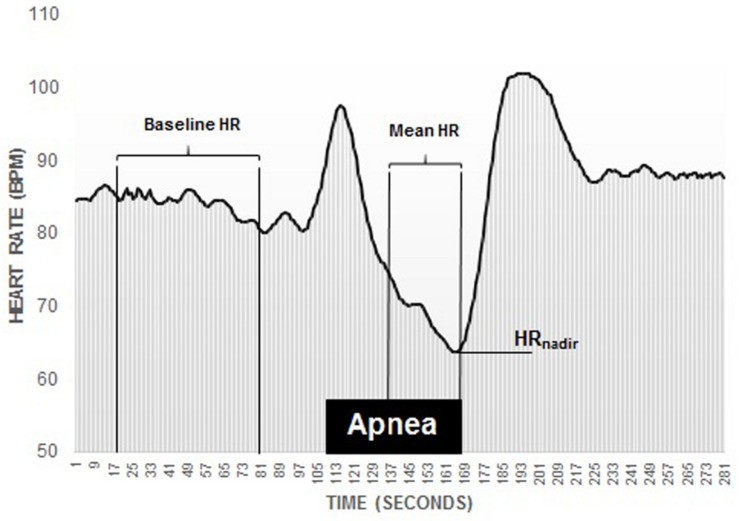
Recording of an individual participants diving bradycardia during 1 min apnea. Note the initial tachycardia, followed by a decline phase. The diving response was defined as the percentage reduction in HR (heart rate) during the period from 30 s into the apnea until the end of the apnea, marked as Mean HR, which was compared to the period 90–30 s before the apnea (baseline).

The magnitude of the diving response was interpreted through the diving bradycardia which was quantified by the apnea-induced HR-reduction from baseline. Baseline HR was computed by calculating the mean HR (bpm) from 90 to 30 s prior to A1 and used as a reference value. To calculate the mean apnea-induced HR-reduction, the mean HR across the apnea duration, minus the first 30 s (A-30) was computed, thus eliminating the initial tachycardia and decline phase (Andersson and Schagatay, 1998). Maximal HR-reduction was also calculated by identifying the HR_*nadir*_ during each apnea. Quantification of the HR-reduction was done by calculating the percentage change between the baseline HR and the mean HR during the apnea (mean HR-reduction) and the HR_*nadir*_ (maximal HR-reduction; Figure 3).

Baseline SaO_2_ was computed by calculating the mean SaO_2_ from 90 to 30 s prior to A1. To account for the circulatory delay from the lung to the finger, the arterial O_2_ desaturation was calculated as the percentage change between baseline SaO_2_ and the SaO_2__*nadir*_ from initiation of apnea until 30 s after apnea.

Measurements of the maximal splenic length (L), width (W), and thickness (T) were used to calculate spleen volume according to the Pilström equation: V_*spleen*_ = Lπ(WT-T^2^)/3.

The formula describes the difference between two ellipsoids divided by two, based on the observed average shape of the spleen (Schagatay et al., 2005; Lodin-Sundström and Schagatay, 2010). The baseline (resting) spleen volume was obtained by averaging the two consecutive maximal values from the 10-min period before the first apnea and changes that occurred during apneas and recovery were calculated by comparing resting volumes with those immediately after apnea and throughout the recovery.

As all participants followed the same ascent profile to 4410 m, LLQ scores could be summed up to this altitude and used in the analysis. Quantification of AMS symptoms throughout the ascent via the accumulated LLQ score minimizes the influence of the many different conditions that mimic AMS, e.g., exhaustion, dehydration, infection, hangover, migraine and hypoglycemia, which all may lead to some degree of headache (Luks et al., 2017). The primary symptom included in the diagnostic criteria of AMS is headache (Roach et al., 1993), which can potentially lead to an incorrect diagnosis. Furthermore, AMS susceptibility was also evaluated by dividing the participants into subgroups, using median split based on individual accumulated LLQ scores: low LLQ group (Lo; LLQ ≤ 6p); medium LLQ group (Me; LLQ 7–12p) and high LLQ group (Hi; LLQ ≥ 13p).

#### Statistical Analysis

Data are reported as mean ± SD, except for LLQ data, which are reported as median (range). Normality distribution of the data was assessed using Kolmogorov–Smirnov and Shapiro–Wilk tests (*p* > 0.05). Differences in HR-reduction, resting spleen volume and contraction, O_2_ desaturation during each apnea, VC, resting HR and resting SaO_2_ (independent variables) between subgroups were evaluated by independent samples *t*-tests. Within-subject’s comparisons on all variables were performed using paired samples *t*-tests with Bonferroni corrections for multiple comparisons. Magnitude of observed effects were estimated using the standardized mean difference (Cohen’s *d*, effect size [ES]), computed as the mean difference divided by the pooled SD. Effect sizes was presented along with 95% confidence intervals (CI). An ES of 0.0–0.3 was considered a small effect, 0.4–0.7 medium and >0.8 a large effect (Lee, 2016). Bivariate multiple correlation tests with Spearman’s rank correlation coefficients (r_*s*_) were conducted to analyze correlation between dependent (LLQ score) and independent variables. Pearson correlation coefficient (r) were used to analyze correlation between parametric variables. A binomial logistic regression using a conditional forward stepwise selection method was performed to analyze the prediction of LLQ score (dependent variable) by HR-reduction, resting spleen volume, VC, arterial O_2_ desaturation during apnea and gender (independent variables) and odds ratio (OR) estimated, with 95% CI. Dichotomization of AMS symptoms in the logistic regression, were established based on median split of LLQ score, into high and low LLQ score (low ≤ 12p; high ≥ 13p). Only one variable: apnea-induced bradycardia during A1, was retained in the regression model. *p* < 0.05 was considered significant and tendencies were denoted *p* < 0.1.

## Results

### LLQ Score and Apnea Duration

Apneic duration for A1 was 56 ± 7 s with seven participants not able complete 60 s. The maximal voluntary apnea durations were 70 ± 16 s (range: 35–97 s) for A2 and 92 ± 25 s (range: 55–151 s) for A3.

While most participants reported only mild AMS symptoms, one subject was evacuated by helicopter from 4410 m due to severe AMS. There was an individual variation in accumulated LLQ (0–22) LLQ points with a median score of 9 at 4410 m. Individual apneic durations did not correlate with LLQ score (NS for all apneas).

### Apnea-Induced Bradycardia

The mean apnea-induced bradycardia was, for A1: 11 ± 12% (HR_*nadir*_: 70 ± 9 bpm), for A2: 13 ± 14% (HR_*nadir*_: 65 ± 9 bpm), and for A3: 10 ± 13% (HR_*nadir*_: 68 ± 9 bpm) (*p* < 0.001). The individual diving bradycardia varied between −34 and +20% and reached baseline resting values during each of the recovery periods (NS).

**FIGURE 4:**
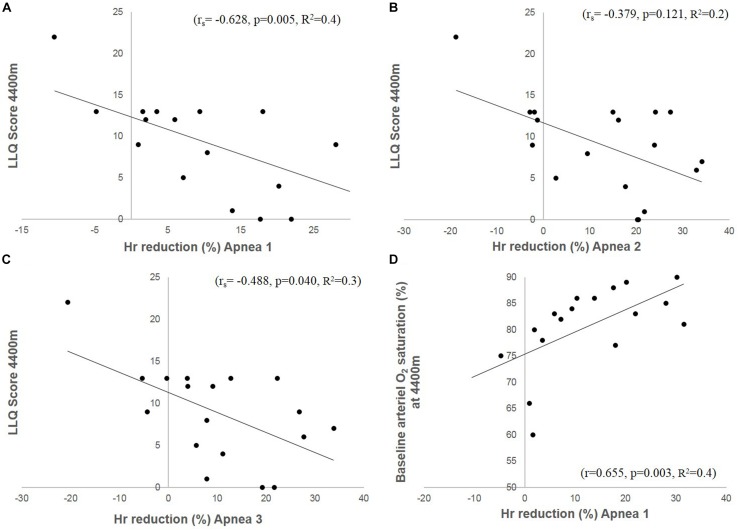
The figures depict correlation plots of accumulated LLQ score at 4410 m and magnitude of the HR (heart rate) reduction (diving bradycardia) during Apnea 1 **(A)**, Apnea 2 **(B)** and Apnea 3 **(C)**. **(D)** Shows HR-reduction (diving bradycardia) during Apnea 1 and SaO_2_ at 4410 m.

The mean apnea-induced bradycardia was negatively correlated with the LLQ score (Figures 4A–C). There was also a correlation between LLQ score and the maximal diving bradycardia for A1 (*r*_*s*_ = −0.542, *p* = 0.02) which became insignificant during A2 (*r*_*s*_ = −0.328, *p* = 0.184) and A3 (*r*_*s*_ = −0.354, *p* = 0.149). The diving bradycardia during apnea was also correlated with SaO_2_ at 4410 m; for A1: *r* = 0.655 (*p* = 0.003; Figure 4D), for A2: *r* = 0.471 (*p* = 0.049) and for A3: *r* = 0.635 (*p* = 0.005). Logistic regression revealed an OR of 1.146 ([CI 1.01–1.30]; *p* = 0.034) for A1.

### Spleen Volume and Contraction During Apnea

**FIGURE 5:**
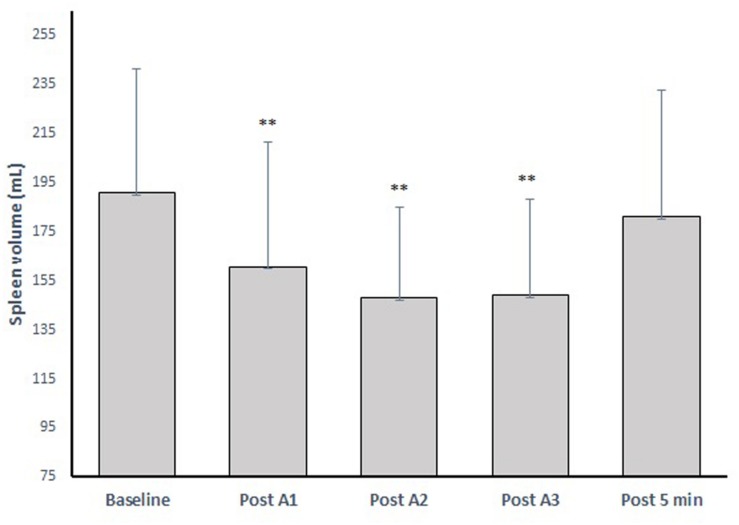
The spleen volume change after three apneic episodes (apnea 1–3) spaced by 2 min of recovery and the volume after 5 min recovery. ^∗∗^ indicates *p* < 0.01 from baseline.

Resting spleen volume was 191 ± 50 mL (range: 86–261 mL), which was reduced after all apneas, to a mean volume of 160 ± 50 mL (−16%) after A1, 147 ± 36 mL (−23%) after A2 and 149 ± 39 mL (−22%) after A3 (Figure 5). Five minutes after A3, the volume had increased to 180 ± 51 mL (−5%; *p* = 0.299).

**FIGURE 6:**
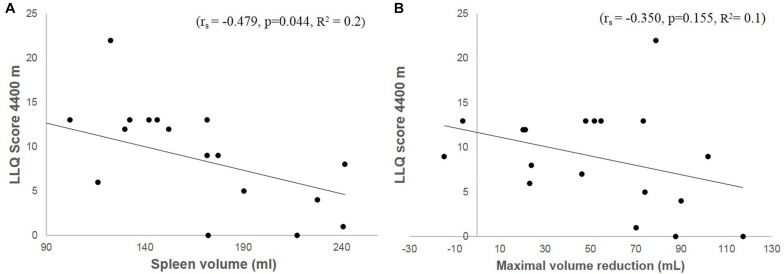
Shows a correlation plot between accumulated LLQ score at 4400 m and baseline (resting) spleen volume **(A)** and maximal volume reduction induced by apnea **(B)** in Kathmandu at 1400 m.

Baseline (resting) spleen volume correlated negatively with LLQ score (*r*_*s*_ = −0.479, *p* = 0.044; Figure 6A). Maximal volume reduction across all apneas correlated with resting volume (*r*_*s*_ = −0.625, *p* = 0.006), but the maximal volume reduction did not correlate with LLQ score (*r*_*s*_ = −0.350, *p* = 0.155; Figure 6B). Neither was there any correlation between LLQ score and relative apnea-induced spleen volume reduction (%).

**FIGURE 7:**
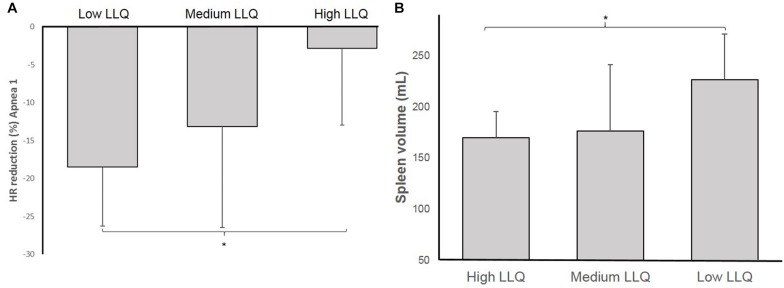
Shows the magnitude of the heart rate (HR) reduction (diving bradycardia) **(A)** and resting spleen volume **(B)** for each of the groups based on accumulated acute mountain sickness (AMS) symptoms score: Low LLQ score, Medium LLQ score, and High LLQ score (^∗^*p* < 0.05).

Comparing the differences in diving bradycardia and spleen volume between sub-groups according to their LLQ score, the diving bradycardia during A1 was more pronounced in the Lo group compared to the Hi group (*p* = 0.014, ES: 1.73 [0.3–2.9]; Figure 7A) and resting spleen volume was greater in the Lo LLQ group compared to Hi LLQ group (*p* = 0.027, ES: 1.59 [0.19–2.75]; Figure 7B).

### Arterial Oxygen Saturation During Apneas

**FIGURE 8:**
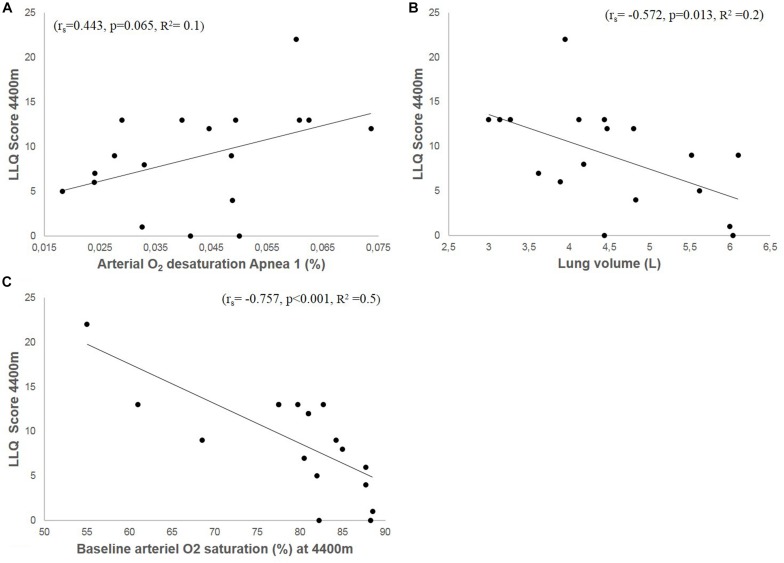
Shows correlation plots of accumulated LLQ score at 4400 m and arterial O_2_ desaturation during apnea 1 **(A)**, vital capacity (VC; **B**), and arterial O_2_ saturation (SaO_2_) at 4400 m **(C)**.

Resting SaO_2_ at low-altitude was 96.3%, which was reduced by 4 ± 1 to 92 ± 2% during A1 (*p* < 0.0001), by 6 ± 4 to 90 ± 4% during A2 (*p* = 0.02) and by 9 ± 6 to 87 ± 6% during A3 (*p* = 0.0014). Arterial O_2_ desaturation during A1 tended to correlate with LLQ score (*r*_*s*_ = −0.443, *p* = 0.065; Figure 8A). During A2, the correlation became insignificant (*r*_*s*_ = −0.352, *p* = 0.152), with no correlation between O_2_ desaturation and LLQ score during A3 (*r*_*s*_ = −0.121, *p* = 0.633).

### Baseline Cardiovascular Variables at 4410 m

Resting SaO_2_ at 4410 m was 79 ± 9% and correlated negatively with LLQ score (*r*_*s*_ = −0.757, *p* < 0.001; Figure 8C). Resting HR at 1400 m was 77 ± 10 and 80 ± 12 bpm at 4410 m (Table 1). The Lo group had higher SaO_2_ at altitude compared to Hi group (Table 1).

**TABLE 1:** Baseline and functional characteristics of the AMS subgroups based on LLQ score.

	***Low LLQ (n6)***	***Medium LLQ (n6)***	***High LLQ (n6)***	***Lo compared to Hi***			***Lo compared to Me***			***Hi compared to Me***		
						
				***p-value***	***ES (d)***	***95% CI***	***p-value***	***ES (d)***	***95% CI***	***p-value***	***ES (d)***	***95% CI***
**Baseline characteristics**												
Age (years)	40 ± 15	46 ± 17	54 ± 7	0.177	1.2	−0.1 to 2.3	0.484	0.4	−0.8 to 1.5	0.365	0.6	−0.6 to 1.7
Gender (f;m)	1;5	2;4	5;1	–	–	–	–	–	–	–	–	–
Height (cm)	178.2 ± 12	173.2 ± 5.5	166.8 ± 4.1	0.064	1.3	0.0 to 2.4	0.376	0.5	−0.6 to 1.7	0.046	1.4	0.1 to 2.6
Weight (kg)	74.3 ± 13	75 ± 12	65.6 ± 5	0.196	0.9	−0.4 to 2.0	0.923	0.1	−1.1 to 1.2	0.120	1.0	−0.2 to 2.6
Body mass index (kg^∗^m^–2^)	23.2 ± 2	24.9 ± 4	23.7 ± 3	0.770	0.2	−1.0 to 1.3	0.342	0.5	−0.7 to 1.6	0.498	0.3	−0.8 to 1.5
**AMS sympoms**												
Accumulated LLQ score [median (range)]	2.5 (0–6)	9.0 (7–12)	13.0 (13–22)	0.0001	–	–	0.0006	–	–	0.02	–	–
**Arterial O_2_ saturation**												
Arterial O_2_ desaturation during A1 (%)	−3.6 ± 1	−4.2 ± 2	−5 ± 1	0.090	1.40	0.05 to 2.54	0.523	0.38	−0.79 to 1.49	0.391	0.51	−0.68 to 1.61
Arterial O_2_ desaturation during A2 (%)	−5.3 ± 3	−5.5 ± 2	−8.7 ± 7	0.293	0.13	−1.26 to 1,01	0.870	0.08	−1.06 to 1.20	0.323	0.25	−0.90 to 1.37
Arterial O_2_ desaturation during A3 (%)	−10.7 ± 7	−6.7 ± 2	−11.1 ± 7	0.965	0.04	−1.17 to 1.09	0.221	0.78	−0.45 to 1.88	0.224	0.85	−0.39 to 1.96
SaO_2_ at 1400 m (%)	95.9 ± 2	96.9 ± 3	96.2 ± 1	0.828	0.19	−1.31 to 0.96	0.458	0.39	−1.50 to 0.78	0.420	0.31	−1.43 to 0.85
SaO_2_ at 4410 m (%)	86.1 ± 3	80.1 ± 6	72.3 ± 11	0.029	1.71	0.28 to 2.88	0.060	1.26	−0.06 to 2.39	0.177	0.81	−0.36 to 1,99
**Heart rate**												
HR at 1400 m (bpm)	75.7 ± 7	76.2 ± 9	84.9 ± 13	0.709	0.88	−1.99 to 0.36	0.969	0.06	−1.19 to 1.07	0.676	0.78	−0.45 to 1.89
HR at 4410 m (bpm)	78.8 ± 14	76.2 ± 14	86.2 ± 9	0.293	0.63	−1.74 to 0.57	0.754	0.19	−0.96 to 1.31	0.178	0.83	−0.40 to 1.94
**Lung volume**												
VC (L)	5.1 ± 1	4.8 ± 1	3.6 ± 1	0.008	1.50	0.12 to 2.64	0.507	0.30	−0.86 to 1.41	0.031	1.20	−0.11 to 2.32
VC (L/cm)	0.028 ± 0.003	0.027 ± 0.004	0.021 ± 0.003	0.0006	2.33	0.73 to 3.58	0.627	0.28	−0.88 to 1.40	0.035	1.70	0.27 to 2.86
**Apnea duration**												
Time A1 (seconds)	56.8 ± 6	52 ± 9	55.5 ± 7	0.725	0.20	−0.95 to 1.32	0.298	0.63	−0.58 to 1.73	0.472	0.43	−0.74 to 1.54
Time A2 (seconds)	74.3 ± 19	61.8 ± 14	66.3 ± 12	0.413	0.91	−0.34 to 2.02	0.232	0.50	−0.68 to 1.61	0.578	0.35	−1.46 to 0.82
Time A3 (seconds)	100.3 ± 35	84.5 ± 14	81.5 ± 17	0.279	0.68	−0.53 to 1.79	0.345	0.59	−0.61 to 1.70	0.748	0.19	−1.31 to 0.96

*Data are presented as Mean ± SD, unless otherwise stated. Effect size (d) along with 95% confidence intervals (CI) is presented for each group.*

### Lung Volume

VC was 4.8 ± 1 L and varied between 3 and 6 L. Individual VC correlated negatively with LLQ score (Figure 8B). VC was also associated with apnea duration (A1: *r* = 0.478, *p* = 0.045; A2: *r* = 0.566, *p* = 0.014; A3: *r* = 0.679, *p* = 0.002). The Lo LLQ group had larger lungs compared to Me and Hi groups (Table 1). The results were similar when lung volumes were expressed related to participants height.

## Discussion

In the present study we investigated the association between apnea-induced physiological responses with the development of AMS symptoms during a trek to mount Everest base camp (EBC). The principal findings were that: (I) the apnea-induced bradycardia for A1 and A3 was negatively associated with accumulated LLQ score; (II) the apnea-induced bradycardia was also associated with SaO_2_ at high altitude (HA); (III) resting spleen volume was negatively associated with accumulated LLQ score with ascent.

### Cardiovascular Diving Response

The finding that the apnea-induced bradycardia is associated with symptoms of AMS suggests that a powerful diving response, which is known to conserve O_2_ and characterizes successful free divers (Schagatay and Andersson, 1998), may also be characteristic of individuals who are tolerant to HA hypoxia. This novel finding provides a promising approach for further studies aiming to develop a simple sea-level test to predict AMS. The association between the diving response and AMS likely result from higher SaO_2_, leading to maintained O_2_ availability for the brain and the heart. This was clearly shown by the association between the apnea-induced bradycardia during both 1 min apnea (A1) and maximal apneas (A3) with accumulated LLQ score, showing that an individual with a pronounced diving response develop less AMS symptoms. However, we did not find any association between the second apnea (A2) and accumulated LLQ score, which was unexpected as we observed a similar magnitude in the HR-reduction for A1 and A3 and a similar O_2_ desaturation during A1. Likely, this may reflect variations in individual HR responses across the apnea series wherein four participants responded with a small HR increase during A2. A noteworthy observation was that resting HR did not correlate with LLQ score; hence, it is the actual apnea-induced bradycardia magnitude that is relevant, with a powerful response reducing the individual risk of developing AMS symptoms.

The metabolic rate decreases during apnea through the CVD, which is strongly associated with apneic diving performance (Schagatay and Andersson, 1998) most likely due to a decrease in O_2_ consumption (Andersson et al., 2002). We suggest that during work at HA, the diving response may have a similar effect. During apnea, the brain and the heart are typically prioritized organs for oxygenation and this situation would also be beneficial at HA. Our investigation also show a less pronounced arterial O_2_ desaturation at HA with a concomitant powerful diving response, which is in agreement with a previous study using simulated altitude, reporting that individuals with a pronounced HR-reduction and maintained levels of SaO_2_ during apnea also maintained high SaO_2_ during simulated altitude (Starfelt et al., 2018). As such, individuals with a powerful diving response during apnea do not only develop less AMS symptoms, but also show less arterial O_2_ desaturation at HA, and thus are less hypoxic and more tolerant to HA hypoxia.

Several investigations have found an association between AMS and SaO_2_ measured at HA (Roach et al., 1998; Karinen et al., 2010; Oliver et al., 2012; Richalet et al., 2012) which is in line with our findings of lower SaO_2_ in participants with more AMS symptoms. For instance, in a large epidemiological study including 1300 participants, Richalet et al. (2012) reported that both resting SaO_2_ and arterial O_2_ desaturation during exercise is greater in participants who develop AMS compared to symptom-free individuals. These findings support the view that SaO_2_ distinguishes those sensitive to altitude hypoxia and those more tolerant.

When participants were divided by AMS symptoms, we found a similar pattern that the group who developed most symptoms also desaturated most, whereas the group who developed least AMS symptoms desaturated less and hence were less hypoxic. Interestingly, we found the same pattern in the three LLQ groups for the diving bradycardia. The group who developed less AMS symptoms had the most pronounced apnea-induced bradycardia, followed by the intermediate LLQ group and high LLQ group. This further shows the contribution of the diving response to maintain high SaO_2_ levels, even in conditions of severe poikilocapnic hypoxia. The low SaO_2_ in AMS susceptible individuals gives credence to an inefficient O_2_-conserving effect through a relative marginal diving bradycardia in the etiology of AMS.

The association between the diving bradycardia and AMS symptoms suggests that the initiating mechanism of the diving response shares a common pathway between HA and apneic diving. At HA, a high HVR is associated with superior performance (Schoene et al., 1984) and with protective properties against AMS (Nespoulet et al., 2012; Richalet et al., 2012). HVR has also been found to have a protective function during apnea, wherein HVR predicts apneic duration and SaO_2__*nadir*_ (Feiner et al., 1995), and apneic divers have higher HVR during poikilocapnic hypoxia compared to non-apneic divers (Costalat et al., 2014), resulting from higher chemoreceptor responsiveness in the former group. However, during apnea the diving bradycardia is not initiated by hypoxia, but by cessation of respiration and augmented by face immersion via stimulation of cutaneous facial cold receptors (Schutema and Holm, 1988). This was also apparent in the present study, although face immersions were not used, thus the magnitude of the apnea-induced bradycardia was almost similar during A1 compared to A2 and A3, which did not cause a severe drop in SaO_2_ during A1. However, PaO_2_ regulates the bradycardia, where decreased PaO_2_ below normal values will cause a more powerful response (Gooden, 1994), which might imply that individuals with a higher chemoreceptor responsiveness during apnea, eliciting a more powerful diving response, may also have a higher HVR at HA.

A powerful diving response may also reflect a dynamic vascular system, involving a general ability of the body to transiently prioritize blood flow to the most important regions. Endothelial function [flow-mediated dilatation (FMD)] is essential for vasoregulation. At HA, preservation of the vascular system through maintained FMD is crucial for O_2_ delivery to vital organs. Redistribution of blood flow through peripheral vasoconstriction occurs at HA during exercise, whereby the body down-prioritizes muscle oxygenation (Calbet et al., 2003) most likely in favor of the most hypoxia-sensitive organs, as occurs during diving. In addition, cerebral blood flow increases at HA in response to low PO_2_, as a means to swiftly shuttle O_2_ to the brain (Ainslie and Subudhi, 2014). Upon acute exposure to HA, FMD is reduced in those who develop AMS compared to asymptomatic individuals, where reduction in FMD occurs well before the onset of AMS with increased arterial O_2_ desaturation (Bruno et al., 2016). We speculate that a pronounced diving response contributes to/reflects a dynamic cardiovascular system at HA, which would reduce O_2_-consumption by prioritizing and redistributing blood flow. This mechanism could have contributed to the higher SaO_2_ observed at HA in symptom-free individuals in the present study, and thereby be protective against AMS.

### Spleen Volume and Contraction

An additional new finding of the present study is that resting spleen volume is negatively associated with AMS symptoms development, i.e., individuals with larger spleens develop less AMS symptoms. Previous investigations have shown that different hypoxic situations stimulate the spleen to contract and release red blood cells (RBCs), both during apnea (Schagatay et al., 2001) and during normobaric poikilocapnic hypoxia (Lodin-Sundström and Schagatay, 2010), resulting in increased circulating RBC which increases the blood gas transportation. The primary driver to induce splenic contraction is hypoxia (Lodin-Sundström and Schagatay, 2010), however, other mechanisms have been proposed, such as hypercapnia (Richardson et al., 2012) and sympathetic activation (Frances et al., 2008), which may be a possible cause for the response which occurred during A1 in the present study, during which SaO_2_ was still broadly maintained above 92%.

We hypothesized that the function to increase blood gas transportation capacity would also be efficient at HA, in which a greater spleen volume and ability to contract could be beneficial in coping with HA hypoxia, by enhancing O_2_-carrying capacity. The findings of an association between individual spleen volume and AMS symptoms supports our hypothesis. The observation is in line with earlier findings in free divers, which suggests that spleen volume was largest in the athletes with the best results in a free diving world championship (Schagatay et al., 2012). Also, native Bajau divers, who are continuously exposed to hypoxia, were recently reported to have larger spleens compared to a native population within close proximity but not exposed to hypoxia associated with apneic diving (Ilardo et al., 2018). We also reported larger spleens in the most hypoxic COPD patients, compared to less hypoxic patients (Schagatay et al., 2015). Furthermore, we found that spleen contraction was enhanced after a HA expedition to the summit of mount Everest (Engan et al., 2014). These studies suggest that spleen volume and function could be affected by training/exposure. However, it cannot be determined in the present study if participants tolerant to AMS had larger spleens due to genetics, or as a result of training. None of the participants in our sample had visited HA (≥3000 m) in the 2 months prior to the trek to EBC.

The mechanism by which a large spleen could be protective against AMS is that a higher circulating Hb would enhance blood O_2_ carrying-capacity, and the larger the spleen, the more RBC can be expelled, which is supported by a strong association between spleen volume and volume contraction in our study. Therefore, an association could also be expected with the maximal volume reduction, however, only a weak tendency was observed in our study. We expected that the participants most sensitive to HA, with the greatest SaO_2_ reductions at altitude, would have a greater volume contraction. However, there were no association between SaO_2_ at HA and the magnitude of spleen volume reduction. On the other hand, we found that the group with least AMS symptoms, had both larger resting spleen volume and maximal spleen volume reduction compared to the group who developed most AMS symptoms, indicating the superior contractile function of the bigger spleens, allowing a greater increase in RBC, which is associated with tolerance to HA hypoxia.

We believe this lack of association between spleen volume reduction and AMS symptoms is partly due to the discrepancy between AMS subgroups, i.e., the spleen volume reduction was smaller in the intermediate group compared to the high LLQ group, which was unexpected. Looking at the group with low AMS symptoms, we observe a resting volume of 226 mL, a maximal volume reduction of 77 mL and a pronounced diving response (18% HR-reduction), a clear protective characteristic of HA tolerant individuals resulting in higher SaO_2_ and subsequently less AMS symptoms. Conversely, the two other groups exhibits smaller and almost similar resting spleen volumes, of 169 and 176 mL, but different volume reduction and levels of SaO_2_. Presumably, the smaller relative contraction of the intermediate group compared to the Hi group is due to other superior means to cope with hypoxia, e.g., a powerful diving response of 13% HR-reduction, leading to less severe arterial O_2_ desaturation at HA. Meanwhile, participants who develop more AMS symptoms, exhibit less functional defense systems against hypoxia, e.g., have a smaller magnitude in apnea-induced bradycardia of <3%, leading to more severe arterial O_2_ desaturation during apnea and at HA. This would thus lead to a stronger/maximal spleen volume contraction, which would thus serve as a second line of defense against hypoxia, and only be triggered maximally if the hypoxia is severe enough. If the spleen were small, however, it would not have as large effect in protecting against hypoxic insult, as the RBC boost into circulation would still be relatively small.

### Lung Volume

We also found that VC was associated with AMS symptoms development, which has been observed previously (Hackett et al., 1982), wherein VC measured in Kathmandu was inversely associated with SaO_2_ at HA and AMS symptoms. This is also in line with previous findings in free divers who reported greater lung volume in elite apneic divers compared to non-apneic divers (Schagatay et al., 2012). A greater lung is a vital part of apneic diving performance, as it leads to greater O_2_ storage capacity, hence increased O_2_ availability during apnea. In regard to altitude sensitivity, individuals with relatively smaller lungs are more likely to desaturate more (Hackett et al., 1982), thus be at increased risk of developing AMS. It has also been reported that lung volume characterizes individuals that tolerate HA hypoxia well. Tibetans, for instance, exhibit both greater VC and forced VC compared to lowland Han Chinese (Gilbert-Kawai et al., 2014), a physiological adaptation brought about generations of continued exposure to HA hypoxia.

### Study Limitations

The current study design is associated with a number of limitations, which should be acknowledged. Although the strength of the study relates to the experimental procedure and establishing a well-functioning laboratory in the field for advanced physiological testing during apnea. A study limitation is that AMS symptoms are self-reported. The LLQ depends solely on individual subjective perception of the occurrence and severity of symptoms (Roach et al., 1993).

Although all participants were in the same trekking group, hiking together along the same ascent profile, it may still be difficult to standardize an exact ascent rate, water and food intake and additional drug use, e.g., ibuprofen to relieve headache, factors which may have effects on AMS symptoms development.

SaO_2_ measurements via pulse oximetry in the field may also have its limitations. This may lead to misinterpretations in certain situations with peripheral vasoconstriction due to cold extremities or hypoxia at HA or during apnea, which may influence the data output (Luks and Swenson, 2011). However, efforts were made to minimize these factors causing measurements error. All measurements were conducted indoors; maintaining peripheral skin temperatures, and by using a pulse oximeter with a light signal indicating when blood flow was sufficient to obtain good data.

Furthermore, the low sample size of the current study may also have affected the outcome of the logistic regression model. Therefore, to continue to develop the prediction of AMS susceptibility through this easy-to use and non-invasive apnea test we propose, these results should be further studied in a larger population.

## Conclusion

We conclude that the apnea-induced bradycardia and resting spleen volume were inversely associated with AMS symptoms development at HA. These novel findings highlights important links between physiological responses to HA and apneic diving which could contribute to furthering the understanding of the great inter-individual differences in susceptibility to AMS. Measuring individual cardiovascular, splenic and hematological responses induced during apnea at sea-level could possibly be an alternative approach to predict AMS susceptibility in native lowlanders prior to ascent.

## Data Availability

The raw data supporting the conclusions of this manuscript will be made available by the authors, without undue reservation, to any qualified researcher.

## Author Contributions

PH planned and organized the laboratory and field study tests and procedures, collected and analyzed the data, and wrote the manuscript. EM planned and organized the laboratory and field study tests and procedures, collected the data, and proofread the manuscript. AS organized the field laboratory, collected the data, and proofread the manuscript. PL collected the data and proofread the manuscript. ES conceived the idea, planned and organized the laboratory and field study tests and procedures, collected the data, wrote the manuscript, and proofread the manuscript.

## Conflict of Interest Statement

The authors declare that the research was conducted in the absence of any commercial or financial relationships that could be construed as a potential conflict of interest.
